# Antimicrobial susceptibilities of *Proteus mirabilis*: a longitudinal nationwide study from the Taiwan surveillance of antimicrobial resistance (TSAR) program

**DOI:** 10.1186/1471-2334-14-486

**Published:** 2014-09-05

**Authors:** Jann-Tay Wang, Pei-Chen Chen, Shan-Chwen Chang, Yih-Ru Shiau, Hui-Ying Wang, Jui-Fen Lai, I-Wen Huang, Mei-Chen Tan, Tsai-Ling Yang Lauderdale, TSAR Hospitals

**Affiliations:** Division of Infectious Diseases, Department of Internal Medicine, National Taiwan University Hospital, 7 Chung-Shan S. Road, Taipei, 10002 Taiwan; National Institute of Infectious Diseases and Vaccinology, National Health Research Institutes, No. 35 Keyan Road, Zhunan, 35053 Taiwan; College of Medicine, National Taiwan University, 1 Jen-Ai Road Section 1, Taipei, 10051 Taiwan

**Keywords:** Drug resistance, Extended-spectrum β-lactamase, AmpC β-lactamase, *Proteus mirabilis*, Clinical and Laboratory Standards Institute (CLSI)

## Abstract

**Background:**

Longitudinal nationwide data on antimicrobial susceptibility in *Proteus mirabilis* from different sources are rare. The effects of the revised Clinical and Laboratory Standards Institute (CLSI) β-lactam breakpoints on susceptibility rates and on detecting extended-spectrum β-lactamase (ESBL) and AmpC β-lactamase-producers in this species are also seldom evaluated. The present study analyzed data from the Taiwan Surveillance of Antimicrobial Resistance program to address these issues.

**Methods:**

Isolates were collected biennially between 2002 and 2012 from 25 to 28 hospitals in Taiwan. Minimum inhibitory concentrations (MIC) were determined by reference broth microdilution method. All isolates with aztreonam, ceftazidime, or cefotaxime MIC ≥ 2 mg/L were checked for the presence of ESBL by CLSI confirmatory test and subjected to ESBL and AmpC β-lactamases gene detection by PCR. Univariate and multivariate analyses were performed.

**Results:**

Between 2002 and 2012, a total of 1157 *P. mirabilis* were studied. Susceptibility to cefotaxime, ceftazidime, and ciprofloxacin decreased significantly during the past decade, from 92.6% to 81.7%, 100% to 95.2%, and 80.1% to 53.8%, respectively (*P* < 0.01). The revised CLSI breakpoints had significant impact on susceptibility to cefazolin (2009 vs. current breakpoints, 71.9% vs. 0.9%) and imipenem (99.8% vs. 55.1%) (*P* < 0.001 for both). However, using the 2014 cefazolin breakpoints for urinary tract infections, 81.2% of the urine isolates were susceptible. Susceptibilities of isolates from different specimen types were mostly similar but outpatient isolates were more susceptible than inpatient isolates. The overall prevalence of ESBL- and AmpC- producers was 8.2% and 4.7%, respectively, but AmpC carriage increased significantly over the years (from 0 to 7.0%, *P* < 0.001). ESBL and AmpC β-lactamase-producers were more likely to be found in elderly and ICU patients. The predominant ESBL and AmpC β-lactamase genes were CTX-M- and CMY- types, respectively.

**Conclusions:**

A significant decrease in susceptibility to 3rd-generation cephalosporins and ciprofloxacin occurred in *P. mirabilis* from Taiwan in the past decade. The prevalence of ESBL remained stable but AmpC β-lactamase-producing *P. mirabilis* increased significantly. Cefotaxime was a better surrogate than ceftazidime for predicting the presence of these β-lactamases. Continuous surveillance on antimicrobial resistance and associated resistance mechanisms in *P. mirabilis* is warranted.

**Electronic supplementary material:**

The online version of this article (doi:10.1186/1471-2334-14-486) contains supplementary material, which is available to authorized users.

## Background

*Proteus mirabilis* belongs to the *Enterobacteriaceae* family with the features of swarming motility and production of urease to generate ammonia [1, 2]. It can be found in soil, water, and the intestinal tract of mammals, including humans. In addition to being a leading cause of urinary tract infections (UTI), *P. mirabilis* can cause respiratory and wound infections, bacteremia, and other infections [1, 2]. Although *P. mirabilis* is usually not a common cause of UTI among immunocompetent individuals, it is an important pathogen among patients with complicated urinary tract, urolithiasis, or long-term urinary catheterization [3]. Patients with UTI caused by *P. mirabilis* usually have alkaline pH urine due to the presence of ammonia resulting in calcium and magnesium crystallization which could in turn lead to obstruction of the lumen of indwelling catheters [4].

*P. mirabilis* was susceptible to cephalosporins and β-lactam/β-lactamase inhibitors. However, strains resistant to β-lactams mediated by acquired β-lactamases emerged in 1990s [5]. Among these β-lactamases, plasmid-borne extended-spectrum β-lactamases (ESBL) and AmpC β-lactamases are most worrisome because they result in resistance to nearly all penicillins and cephalosporins and can spread among different species of *Enterobacteriaceae* [6]. Several other studies have shown that ESBL and AmpC β-lactamase-producing *P. mirabilis* isolates could lead to clonal spread and then cause intra-hospital, regional, and continent-wide outbreaks [7, 8]. Treatment failure and clinical mortality are also more likely to occur in patients infected with ESBLs-producing *P. mirabilis* [9], which has been attributed to inadequate empirical therapy. The emergence and global spread of carbapenemase-producing *Enterobacteriaceae* (CPE) in recent years, especially isolates carrying genes encoding KPC (*Klebsiella pneumoniae* carbapenemase) and NDM (New Delhi metallo-β-lactamase) carbapenemases, have further compromised treatment options and added to the crisis of antimicrobial resistance [10, 11]. However, recent studies from different regions have found the prevalence of carbapenemase-producing *P. mirabilis* remained low [12–14].

Studies from the United States, Canada, United Kingdom, and other European countries revealed that susceptibility of *P. mirabilis* isolated from different sources can vary widely. For example, susceptibility to β-lactam/β-lactamase inhibitors (ampicillin/sulbactam or amoxicillin/clavulanate), ciprofloxacin, and third generation cephalosporins (cefotaxime, ceftriaxone, or ceftazidime) ranged 74 to 94%, 60 to 90%, and 90 to 99%, respectively, depending on patient population and specimen type [12, 15–18]. Therefore, surveillance on the *in vitro* susceptibilities of *P. mirabilis* in each region is clinically relevant and important.

In addition, the Clinical and Laboratory Standards Institute (CLSI) revised the interpretive criteria on several β-lactams for *Enterobacteriaceae* in recent years. In 2010, CLSI lowered the aztreonam and 3rd-generation cephalosporin breakpoints for *Enterobacteriaceae* to facilitate the identification of isolates expressing ESBLs and/or AmpC β-lactamases. Breakpoints for different carbapenems were updated in June 2010 and in 2012. The most recent revision for cefazolin breakpoints occurred in 2011 and in 2014 (for isolates from urine only). Cefepime breakpoints were also revised in 2014. These changes are summarized in the 2014 CLSI M100-S24 document [19]. Of noteworthy also is that *Proteus* spp. can have naturally higher imipenem MICs [19, 20]. Therefore, the new CLSI carbapenem breakpoints can have significant effect on the rate of imipenem susceptibility in *P. mirabilis*. To date, the impact of these changes on the susceptibilities to 3rd-generation cephalosporins and the correlation with carriage of ESBL and/or AmpC β-lactamases, and the effect on cefazolin and carbapenem susceptibility in *P. mirabilis* have seldom been discussed [21].

In Taiwan, *P. mirabilis* remains an important pathogen causing UTI among patients with urolithiasis and urinary catheters in both community and healthcare settings [22]. However, national data on the susceptibilities of *P. mirabilis* from different sources are limited. The Taiwan Surveillance of Antimicrobial Resistance (TSAR) is a biennial nationwide program conducted at the National Health Research Institutes (NHRI) [23]. The present study analyzed the TSAR data from period III (2002) to VIII (2012) to address the above issues.

## Methods

### Isolate collection

**Figure 1:**
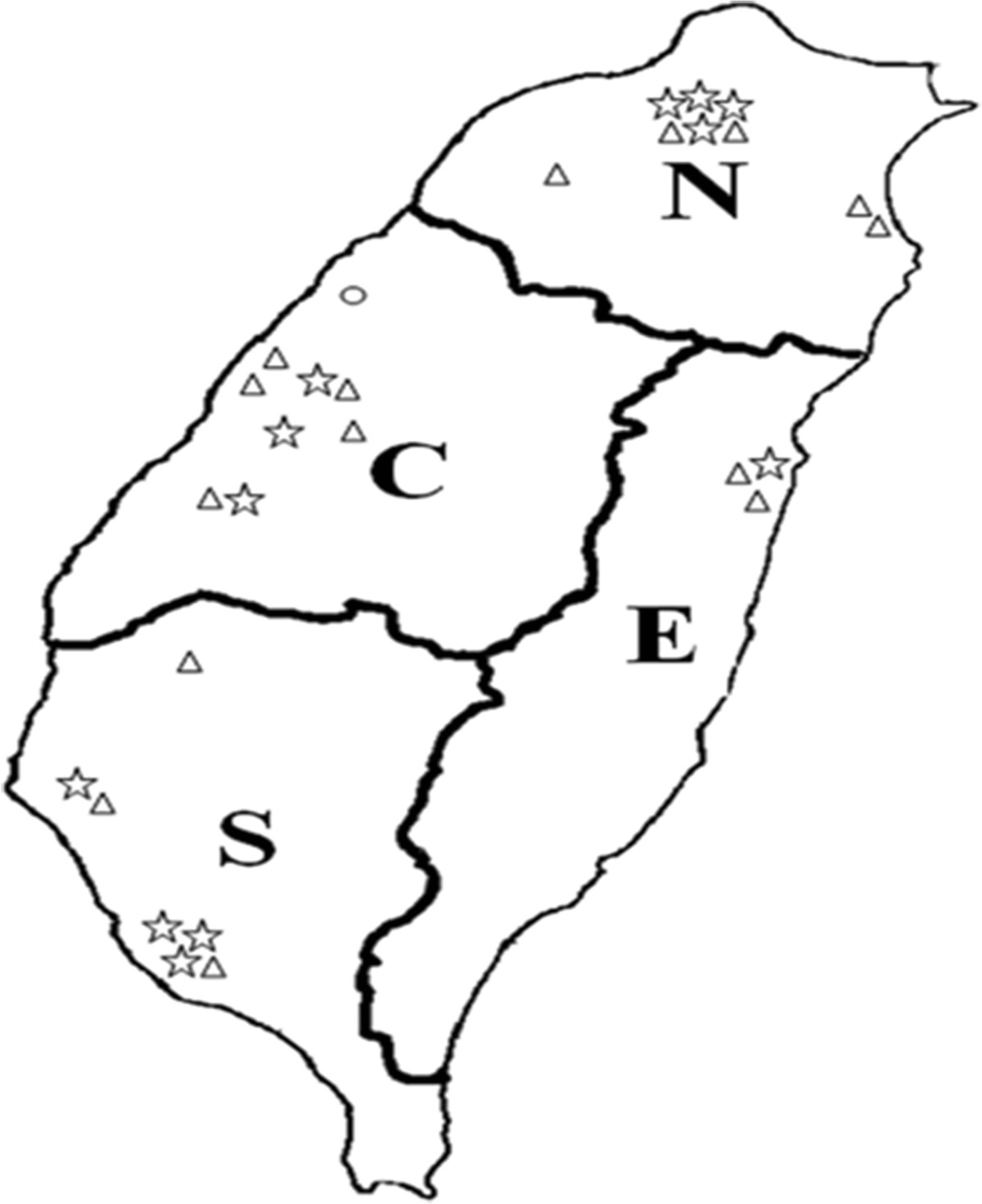
**Distribution of hospitals that participated in the Taiwan Surveillance of Antimicrobial Resistance (TSAR) program from 2002 (TSAR III) to 2012 (TSAR VIII).** The proximate locations of the hospitals are shown in each region (N, north; C, central; S, South; E, East). Taiwan is a mountainous island and the majority of the people live in the most densely populated western part (north, central and south regions) while the eastern part is the least populated region. Hospital type: star, medical center; triangle, regional hospital; circle, local hospital, which participated in TSAR VIII (2012) only.

*P. mirabilis* isolates were collected as part of the TSAR program. Isolates were collected biennially between 2002 and 2012 (corresponding to TSAR III – VIII). For TSAR III to VII, isolates were collected from the same 26 hospitals (11 medical centers and 15 regional hospitals) except in 2008, when one hospital did not participate. In TSAR VIII (2012), isolates were collected from 25 of these 26 hospitals and two additional hospitals. These hospitals are located in the four geographic regions of Taiwan and all are general hospitals (Figure 1). The isolates were collected between July and September during the collection year and the collection process has been described previously [23]. Isolates were collected sequentially without specifying species to be collected. All isolates were stored at - 70°C in bead-containing Microbank cryovials (PRO-LAB Diagnostics, Austin, TX, USA) (for 2002 to 2006) or glycerol (20%) containing trypticase soy broth (for 2008 to 2012). The bacterial isolates were recovered from clinical samples taken as part of standard care. The study was approved by the Research Ethics Committee of National Health Research Institutes (NHRI) (EC960205 and EC1010602-E).

### Isolate identification

Isolates reported as *P. mirabilis* by hospitals were subcultured to blood agar and MacConkey agar plates for purity check and to confirm species identification at NHRI. Species identification was based on colony morphology and conventional biochemical reactions. For isolates with colony morphology or any biochemical reactions not typical of *P. mirabilis*, either Vitek I (prior to 2008) or Vitek II (2008 to 2012) GN cards were used (bioMérieux, Marcy l’Etoile, France).

### Antimicrobial susceptibility testing (AST)

Minimum inhibitory concentrations (MICs) were determined by the broth microdilution method following the guidelines of the manufacturer and Clinical and Laboratory Standards Institute (CLSI) [24]. Sensititre custom-designed plates were used from 2002 to 2008, and the standard GNX2F plates were used in 2010 and 2012 [ThermoFisher Scientific (formerly Trek Diagnostics), East Grinstead, UK]. All isolates were subcultured twice on sheep blood agar plates from -70°C prior to AST. Quality control was performed on each day of test with *Escherichia coli* ATCC 25922, *E. coli* ATCC 35218, and *Pseudomonas aeruginosa* ATCC 27853. The agents below were tested on isolates from all years: cefotaxime, ceftazidime, cefepime, aztreonam, imipenem, amikacin, gentamicin, ciprofloxacin, and trimethoprim/sulfamethoxazole (SXT). The following agents were tested on isolates from 2002 to 2008: ampicillin, amoxicillin/clavulanate, piperacillin, cefazolin, cefuroxime, cefoxitin; nitrofurantoin. In addition, ertapenem and meropenem were tested on isolates from 2010 and 2012. Interpretive criteria are based on the 2014 CLSI breakpoints. The impact of CLSI breakpoint revisions on susceptibilities to aztreonam, cefazolin, cefepime, cefotaxime, ceftazidime, ertapenem, imipenem, and meropenem are also compared using the current breakpoints and those of 2009, the year prior to different revisions [25].

### Determination of carriage of ESBL and/or AmpC β-lactamases genes

All isolates with aztreonam, ceftazidime, or cefotaxime MIC ≥ 2 mg/L were tested for the presence of ESBL by CLSI confirmatory test using cefotaxime and ceftazidime with and without clavulanic acid [25]. These isolates were also subjected to ESBL and/or AmpC β-lactamases gene detection by PCR. For each tested isolate, DNA extraction was performed using the following procedures. Three to five colonies were lightly picked from fresh overnight culture plate to suspend in 150 μl AE buffer. The suspension was heated at 95°C for 15 min then centrifuged at 1000 g for 10 min to remove cellular debris, after which 100 μl of the supernatant was transferred to a new vial. The DNA preparation was stored at -20°C and used as template for subsequent amplifications. Multiplex PCR was used to detect the relevant genes following previously described primers and protocols [26, 27].

### Data analysis

Susceptibility interpretation analysis was made using the WHONET software [28]. Duplicate isolates were excluded from analysis. The chi-square test was used for trend analysis on susceptibility to different agents over the years. Significance of differences in rates of susceptibility was tested by the *χ*2 test or Fisher’s exact test (if the number was less than 10). The variable tested included β-lactam agents having old and revised CLSI breakpoints, and on isolates from different specimen types (blood, urine, pus/abscess, sputum) and patient locations [inpatients: intensive care units (ICU) or non-ICU, and outpatients]. Multivariable logistic regression analysis was performed to assess the variables (including study year, specimen type, patient age group, and patient location) among ESBL or AmpC β-lactamase -producers vs. -non-producers. All analyses were performed using SAS 9.2 (SAS Institute, Cary, NC, USA). A 2-tailed *P* value less than 0.05 was considered statistically significant.

## Results

### Isolates

A total of 1,157 *P. mirabilis* isolates were collected by the TSAR program with 176, 186, 205, 219, 185, and 186 isolates from 2002, 2004, 2006, 2008, 2010, and 2012, respectively. The most common specimen type was urine, accounting for 49.4% (571), followed by pus/abscess (233, 20.1%), blood (158, 13.7%), sputum (133, 11.5%), and others (62, 5.4%). The age of the source patients was known in 1110 patients. The mean age of the patients was 62.8 ± 24.7 years, with 9.2% being pediatric patients (≤18 years), 29.6% being adult (19–64 years), and 61.3% being elderly (≥65 years). Most isolates were from inpatients (65.2%; 53.6% in general ward, and 11.6% in ICU).

### Susceptibility to different antimicrobial agents over the years and the impact of different CLSI breakpoints

**Figure 2:**
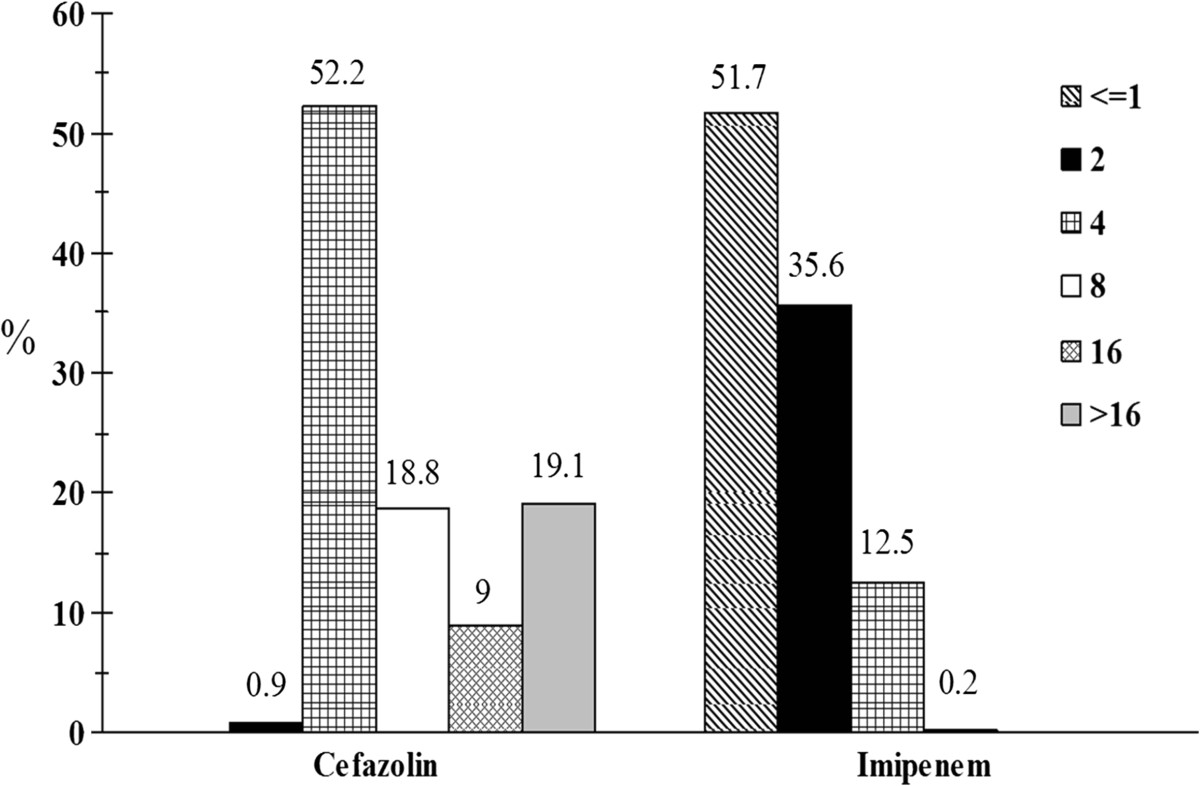
**MIC distributions of cefazolin and imipenem in** ***Proteus mirabilis*** **.** Data for cefazolin and imipenem are from 786 and 1157 isolates, respectively (cefazolin was not tested in 2010 and 2012) in the Taiwan Surveillance of Antimicrobial Resistance (TSAR) 2002 to 2012 program. The susceptible, intermediate, and resistant breakpoints (2009 vs. current) are < = 8, 16, and > =32 vs. <= 2, 4, and > =8 mg/L for cefazolin; and < = 4, 8, and > =16 vs. <= 1, 2, and > =4 mg/L for imipenem. Numbers on top of the bars represent % of isolates with that MIC value.

The susceptibilities of the 1157 isolates to various antibiotics by year are presented in Table 1. Significant decrease in susceptibilities over the years (from 2002 to 2012) to cefotaxime (from 92.6% to 81.7%), ceftazidime (from 100% to 95.2%), and ciprofloxacin (from 80.1% to 53.8%) occurred. The revised CLSI breakpoints impacted significantly (overall susceptibility 2009 vs. current, *P* < 0.001) on susceptibilities to cefazolin (71.9% vs. 0.9%), cefotaxime (95.2% vs. 85.7%), and imipenem (99.8% vs. 55.1%). The MIC distribution of cefazolin and imipenem are shown in Figure 2 to illustrate how the breakpoint change affected the susceptibility rate. Using the 2009 vs. current breakpoints, 9% vs. 52.2% and 19.1% vs. 46.9% of the isolates had cefazolin MIC in the intermediate (4 mg/L) and resistant (> = 8 mg/L) range, respectively. For imipenem, the revised MIC breakpoints resulted in 35.6% and 12.7% of the isolates in the intermediate (2 mg/L) and resistant (> = 4 mg/L) category, respectively. The revised breakpoints did not have significant effect on the susceptibility to ceftazidime (98.4 vs. 97.2%), aztreonam (99.5 vs. 99.3%), ertapenem (100 vs. 99.7%), and meropenem (all 100%) (Table 1).

**Table 1:** **Antimicrobial susceptibilities (%) of** ***Proteus mirabilis*** **by study year, 2002-2012**

Antimicrobial agents^a^	2002 (n = 176)	2004 (n = 186)	2006 (n = 205)	2008 (n = 219)	2010 (n = 185)	2012 (n = 186)	*P* ^b^	total (n = 1157)
β-lactams:								
Amoxicillin/CA^c^	91.4	74.7	86.8	83.6	NT	NT		84.6
Ampicillin	33.3	32.3	31.7	39.3	NT	NT		34.3
Aztreonam_2009	99.4	99.5	98.5	100	100	99.5		99.5
Aztreonam	99.4	98.9	98.5	100	100	98.9		99.3
Cefazolin_2009	73.6	64.0	74.2	75.3	NT	NT		*71.9* ^d^
Cefazolin	0.6	0	2.4	0.5	NT	NT		*0.9*
Cefuroxime	92.5	83.3	85.9	88.6	NT	NT		87.5
Cefotaxime_2009	95.5	88.2	96.6	94.9	98.4	97.3		*95.2*
Cefotaxime	92.6	84.4	86.8	88.5	80	81.7	0.003	*85.7*
Ceftazidime_2009	100	99.5	99	98.2	97.8	95.7	0.001	98.4
Ceftazidime	100	98.4	98.1	96.4	95.6	95.2	0.001	97.2
Cefoxitin	96	94.1	94.6	95	NT	NT		94.9
Cefepime	97.2	91.4	97.6	99.1	100	98.9		97.4^e^
Ertapenem_2009	NT	NT	NT	NT	100	100		100
Ertapenem	NT	NT	NT	NT	100	99.5		99.7
Imipenem_2009	100	100	99.5	99.5	100	100		*99.8* ^*d*^
Imipenem	34.7	16.1	56.6	43.4	92.4	67.2		*51.7*
Meropenem	NT	NT	NT	NT	100	100		100
Piperacillin	48.9	44.6	37.6	43.3	NT	NT		43.4
Non β-lactams:								
Amikacin	92.6	88.2	89.8	90.4	90.3	88.7		90
Gentamicin	59.1	60.8	54.2	62.1	55.1	54.3		57.7
Ciprofloxacin	80.1	70.3	68.3	69.9	70.3	53.8	< 0.001	68.7
TMP/SMX (SXT)^c^	35.8	33.3	29.8	37	36.8	31.7		34.1
ESBL prevalence	5.1	10.2	10.7	6.9	5.4	10.8		8.2
AmpC prevalence	0	3.8	2.9	5.0	9.2	7.0	< 0.001	4.7

^a^Susceptibility results are based on the current CLSI breakpoints [19]. For agents with breakpoint revision in recent years, results of the 2009 CLSI criteria are also shown for comparison [25].

^b^Chi-square for trends. Only statistically significant results are shown.

^c^Amox/CA, Amoxacillin/clavulanic acid; TMP/SMX (SXT), trimethoprim/sulfamethoxazole.

^d^Italicized, significant difference (*P* < 0.001) in susceptibility rates using old and revised breakpoints.

^e^Cefepime results include 3.5 % (41 isolates) in the SDD (susceptible dose dependent) category.

### Susceptibility of isolates from different sources

Some variations in rates of susceptibility to individual agents existed for isolates from different specimen types and patient locations (Table 2). By specimen types, isolates recovered from sputum had significantly lower susceptibility to cefazolin (by CLSI 2009 criteria), piperacillin, and trimethoprim/sulfamethoxazole compared to other specimen types. For urine isolates, susceptibility to cefazolin differed significantly using the CLSI 2009 and 2014 updated UTI criteria (81.2 vs. 74.3%, *P* = 0.016) (Table 2). Isolates from outpatients had significantly higher rates of susceptibility to ampicillin, amoxicillin/clavulanate, cefazolin (by CLSI 2009 criteria), cefuroxime, cefotaxime, ciprofloxacin, gentamicin, and piperacillin.

**Table 2:** **Antimicrobial susceptibilities of** ***Proteus mirabilis*** **by specimen types and patient locations**

Antimicrobial agents^a^	Specimen types^b^				Patient locations		
	Urine (n = 571)	Pus/abscess (n = 233)	Blood (n = 158)	Sputum (n = 133)	ICU (n = 134)	Non-ICU (n = 618)	Outpatients (n = 401)
β-lactams:							
Amoxicillin/CA^c^	83.2	87.1	88.9	76.9	*72.3* ^*d*^	83.9	88.9
Ampicillin	33.1	36.2	44.4	25.3	33.0	31.7	*40.2*
Cefazolin_2009	74.3	73.6	77.8	*50.6*	58.5	69.2	*81.6*
Cefazolin	1.2	1.2	0	0	1.1	0.7	1.2
Cefazolin_UTI^e^	81.2	-	-	-	*64.7*	79.5	86.5
Cefuroxime	86.9	91.4	90	80.2	78.7	86.9	*92.0*
Cefotaxime	85.6	89.2	84.8	80.3	80.6	84.6	*89.3*
Ceftazidime	98.1	97.9	95.6	94.9	96.3	97.2	97.5
Cefoxitin	94.1	96.9	94.4	94.5	91.5	95.1	96.2
Cefepime^f^	97.0	98.3	99.4	95.6	95.5	97.1	98.5
Imipenem_2009	100	100	100	100	99.3	100	100
Imipenem	34.7	16.1	56.6	43.8	53.0	50.2	53.8
Piperacillin	41.4	51.2	47.8	*29.7*	41.5	38.2	*52.7*
Non β-lactams:							
Amikacin	90.5	93.6	87.3	84.7	85.1	89.8	92
Gentamicin	59.5	63.5	58.9	35.8	52.2	53.9	*65.8*
Ciprofloxacin	69.2	70.8	67.1	64.2	62.7	66.5	*74.6*
TMP/SMX (SXT)^c^	33.1	40.3	34.8	*24.8*	35.1	31.9	37.4

^a^Susceptibility results are based on the current CLSI breakpoints [19]. For cefazolin and imipenem, results of the 2009 CLSI criteria are also shown for comparison [25]. Other agents with overall susceptibility >99% (aztreonam, ertapenem, meropenem) are not shown.

^b^Data on miscellaneous other specimen types (n = 63) are not shown.

^c^Amox/CA, Amoxacillin/clavulanic acid; TMP/SMX (SXT), trimethoprim/sulfamethoxazole.

^d^Italicized, significant difference in susceptibility rate compared to other subgroups.

^e^Result of urine isolates are shown using the 2014 UTI breakpoints [19].

^f^Cefepime results include SDD (susceptible dose dependent) category.

### Prevalence of ESBL and AmpC β-lactamases genes

There were 172 isolates with ESBL phenotype (aztreonam, ceftazidime, and/or cefotaxime MICs ≥ 2 mg/L). Among them, 95 carried ESBL genes and 54 carried AmpC genes including 7 that carried both ESBL and AmpC genes. For the isolates carrying ESBL genes, 92 carried CTX-M-type genes, two carried SHV-type genes, and one carried both SHV and CTX-M genes. For the isolates carrying AmpC genes, 48 carried CMY-type genes, and 6 carried DHA-type genes. By Chi-square for trends, the prevalence of ESBL did not increase significantly during the study period (5.1 – 10.8%, *P* = 0.607; Table 1). However, the prevalence of AmpC β-lactamases gene carriage increased significantly (from 0 to 7.0%, *P* <0.001; Table 1).

### Susceptibilities of isolates carrying ESBL and AmpC β-lactamases genes

Using the current CLSI breakpoints, none of 142 isolates with either ESBL or AmpC gene was susceptible to cefotaxime [sensitivity, 100%; specificity, 97.5%; positive predictive value (PPV), 86.1%; negative predictive value (NPV), 100%]. However, 79.6% of the 142 isolates were susceptible to ceftazidime (sensitivity, 20.4%; specificity, 99.7%; PPV, 68.8%; NPV, 90.0%) and 95.8% were susceptible to aztreonam (sensitivity, 4.2%; specificity, 99.5%; PPV, 75%; NPV, 88.2%). Compared to non-ESBL phenotype and ESBL- and AmpC- negative isolates, ESBL and AmpC β-lactamase-producing isolates had significantly lower rates of susceptibility to amikacin (28.4% - 57.1% vs. 97.8%), cefotaxime (0 vs. 99.7%), and ciprofloxacin (6.8% - 42.9% vs. 75.9%) (Table 3). Of note, near 40% of the ESBL positive isolates had cefepime MIC in the susceptible dose dependent (SDD) range compared with 6.4% of the AmpC-positive only isolates (Table 3).

**Table 3:** **Susceptibilities to key agents among the** ***Proteus mirabilis*** **isolates with and without ESBL or AmpC β-lactamases genes**

Antimicrobial agent^a^	Susceptibility (%)			
	ESBL(+)/AmpC(+)	ESBL(+)/AmpC(-)	ESBL(-)/AmpC(+)	Non-ESBL phenotype & ESBL(-)/AmpC(-)
	(n = 7)	(n = 88)	(n = 47)	(n = 1015)
Amikacin	57.1	28.4	40.4	97.8
Aztreonam	100	94.3	97.9	99.8
Cefepime (S/SDD)^b^	42.85/42.85	29.5/38.6	91.5/6.4	99.9/0.1
Cefotaxime	0	0	0	99.7
Ceftazidime	71.4	94.3	53.2	99.7
Ciprofloxacin	42.9	6.8	34.0	75.9
Ertapenem	100 (n = 4)	100 (n = 26)	100 (n = 26)	99.7 (n = 315)
Imipenem_2009	100	100	100	99.9
Imipenem	42.9	45.5	66.0	51.7

^a^Susceptibility results are based on the current CLSI breakpoints [19]. For imipenem, results of the 2009 CLSI criteria are also shown for comparison [25]. All were susceptible to meropenem.

^b^Cefepime results are shown in susceptible and SDD (susceptible dose dependent) categories.

### Factors associated with ESBL and AmpC β-lactamases gene carriage

Factors associated with carriage of ESBL included age and patient location using univariate analysis (Table 4). By multivariate analysis, age [elderly vs. adult patients; odds ratio (OR), 3.85; 95% confidence interval (95% C.I.), 2.02–7.33; *P* <0.001] and patient location (ICU vs. outpatients; OR, 1.99; 95% C.I., 1.04–3.84; *P* = 0.039) remained independent factors associated ESBL genes carriage. Age (elderly vs. adult patients; OR, 2.22; 95% C.I., 1.06–4.62; *P* = 0.034) was also a sole factor significantly associated with AmpC β-lactamase gene carriage by both univariate and multivariate analyses.

**Table 4:** **Univariate analysis for factors associated with carriage of ESBL and AmpC β-lactamases genes in** ***Proteus mirabilis***

Variables	ESBL				AmpC β-lactamases			
	Odds ratio	95% confidence interval		*P*	Odds ratio	95% confidence interval		*P*
		Lower	Upper			Lower	Upper	
Study year (using TSAR VIII [2012] as baseline)^a^								
TSAR III (2002)	0.45	0.20	1.01	0.053	-	-	-	-
TSAR IV (2004)	0.94	0.49	1.83	0.866	0.52	0.20	1.34	0.174
TSAR V (2006)	0.99	0.53	1.89	0.995	0.40	0.15	1.08	0.070
TSAR VI (2008)	0.61	0.30	1.23	0.167	0.70	0.31	1.61	0.406
TSAR VII (2010)	0.47	0.22	1.04	0.064	1.35	0.63	2.86	0.438
Age groups (using adult as baseline)^b^								
Pediatric patients	0.29	0.04	2.24	0.233	1.07	0.29	4.05	0.916
Elderly patients	4.01	2.11	7.63	<0.001	2.22	1.06	4.62	0.034
Specimen types (using blood as baseline)								
Urine	0.90	0.48	1.69	0.745	0.89	0.40	2.02	0.788
Sputum	0.56	0.25	1.24	0.153	0.75	0.28	2.00	0.569
Pus/abscess	1.56	0.74	3.26	0.241	1.48	0.57	3.85	0.426
others	0.97	0.33	2.83	0.956	0.33	0.04	2.69	0.300
Patients’ location (using outpatient as baseline)								
Non-ICU	2.46	1.29	4.70	0.006	1.07	0.41	2.77	0.890
ICU	2.23	0.99	4.98	0.051	1.21	0.66	2.21	0.545

^a^There were no AmpC β-lactamase gene positive isolates in TSAR III (2002).

^b^Definition of age groups for pediatric, adult, and elderly patients was < = 18, 19–64, and > =65 year olds, respectively.

## Discussion

This multicenter longitudinal surveillance revealed significant decreased susceptibility to cefotaxime, ceftazidime, and ciprofloxacin occurred in *P. mirabilis* from Taiwan in the past decade. In addition, compared to recent reports from the United States, Canada, and United Kingdom, our results showed that *P. mirabilis* in Taiwan have lower rates of susceptibility to cefotaxime (85.7% vs. > 97% in US, Canada, and UK) and gentamicin (57.7% vs. > 90%) [12, 15–17]. Susceptibility to ciprofloxacin was also much lower than rates found in the United States and Canada (68.7% vs. > 80%) [16, 17].

The lower rate of cefotaxime-susceptibility in our isolates might be due to a higher prevalence of ESBL- and/or AmpC β-lactamase- producers. Our overall 8.2% ESBL rate is higher than the <5% reported on *P. mirabilis* from different sources in the United States [15, 16, 29]. In contrast to ESBL-producers, data on changes in AmpC prevalence in *P. mirabilis* are scarce. The increase of AmpC β-lactamase-producers over the years observed in the present study may be due to clonal spread and horizontal gene transfer. The lower and decreased susceptibility to ciprofloxacin over the study periods might be due to increased consumption of fluoroquinolones in Taiwan in recent years [30].

Although the prevalence of ESBL-producers remained stable, AmpC β-lactamase-producers increased significantly over the study years. Whether this phenomenon was due to clonal spread or horizontal gene transfer requires further study. Most ESBL genes were CTX-M types (94.8%). The predominant AmpC β-lactamases genes were CMY-types (88.9%), with the rest being DHA-type. Studies from Taiwan on other species of *Enterobacteriaceae* have found CTX-M-type ESBL and CMY-type and DHA-type AmpC β-lactamases to be prevalent, including *Escherichia coli*, *Klebsiella pneumoniae*, and *Enterobacter* spp. [14]. Of note, there was only one isolate with non-susceptibility to ertapenem (MIC = 1 mg/L). Therefore, carbapenemases do not currently appear to be prevalent in *P. mirabilis* in Taiwan.

Our results also showed that the revised CLSI breakpoints have significant impact on susceptibility to cefazolin (2009 vs. current breakpoints, 71.9% vs. 0.9%), cefotaxime (95.2% vs. 85.7%), and imipenem (99.8% vs. 51.8%) in *P. mirabilis* (*P* < 0.001). The marked decrease in susceptibility to cefazolin is due in part to a large number (52.2%) of isolates falling into the immediate category (MIC 4 mg/L). A study from Canada also showed similar MIC distribution in *P. mirabilis*, with 4.2% and 63.6% of isolates in the susceptible and intermediate category, respectively [17]. However, using the 2014 UTI interpretive criteria for cefazolin, 81.2% of the urine isolates in our study were susceptible. This information can be helpful in therapy selection for uncomplicated UTIs.

The large difference in imipenem susceptibility when using different breakpoints (2009 vs. current, 99.8% vs. 51.8%) is because of the high percentages of *P. mirabilis* isolates having imipenem MICs of either 2 or 4 mg/L, which were considered susceptible by the old criteria but intermediate and resistant, respectively, by the new criteria. It is well known that *P. mirabilis* can present with higher MICs against imipenem compared to other carbapenem agents [19]. In fact, among the 373 *P. mirabilis* isolates that had ertapenem, imipenem, and meropenem tested in our study, 76 had imipenem MIC of either 2 or 4 mg/L. These 76 isolates all had meropenem MIC < = 1 mg/L, 75 of which had ertapenem MIC < = 0.25 mg/L and only one isolate had ertapenem MIC 1 mg/L (data not shown). Therefore, applying the revised CLSI criteria would result in significantly fewer carbapenem-susceptible *P. mirabilis* being reported. Similar results have been found on *P. mirabilis* isolated from ICUs in Taiwan [21]. In the European Committee on Antimicrobial Susceptibility Testing MIC distribution database, 63.8% of the tested *P. mirabilis* (n = 15852) had an imipenem MIC < = 1 mg/L, yet 97.8% had < = 4 mg/L [31]. In two recent studies using the revised breakpoints, low rates of susceptibility to imipenem in *P. mirabilis* (9% and 26.5%) were noted [15, 16]. It has been reported that susceptibility of *P. mirabilis* to carbapenems should be determined by ertapenem, meropenem, or doripenem [21, 32]. Our study echoed this suggestion.

CLSI lowered the susceptibility breakpoints of *Enterobacteriaceae*, including *P. mirabilis*, for 3rd-generation cephalosporins in 2010 to facilitate the identification of isolates having ESBLs and/or AmpC β-lactamases [33]. Using the revised breakpoints, none of the ESBL and/or AmpC positive isolates was susceptible to cefotaxime, yet 53.2% to 94.3% remained susceptible to ceftazidime. Therefore cefotaxime is a better predictor for ESBLs and/or AmpC β-lactamase-producers than ceftazidime in terms of sensitivity (100% vs. 20.4%) and PPV (86.1% vs. 68.8%). This echoed the findings of prior studies on other species of *Enterobacteriaceae* [34, 35]. The higher sensitivity and PPV of cefotaxime reflects the presence of CTX-M since it hydrolyzes cefotaxime more efficiently than ceftazidime [36]. Of interest also is that using the 2014 revised cefepime breakpoints, around 40% of the ESBL-producers had cefepime MIC in the susceptible dose dependent range, indicating that higher dosing regimens are needed if cefepime was used for these isolates [19].

Independent factors associated with the presence of ESBL genes were age (elderly patients) and location (ICU) of source patients. The only independent factor associated with the presence of AmpC β-lactamase genes was age (elderly patients) of source patients. Both of these two factors implied higher prior antibiotic use and/or more broad-spectrum antibiotic exposure, which in turn can result in acquisition of drug-resistance genes.

One limitation of the present study is that isolates were collected biennially during a three months period. However, our isolates were from 28 hospitals located in all four regions of Taiwan, 25 of which participated in all 6 rounds of TSAR between 2002 and 2012. These 28 hospitals included 12 medical centers, 15 regional hospitals, and one local hospital. Therefore, the results presented here are a representation of the total number of *P. mirabilis* in Taiwan.

## Conclusion

This multicenter surveillance revealed decreased susceptibility of *P. mirabilis* in Taiwan to some broad spectrum antibiotics, including 3rd-generation cephalosporins and ciprofloxacin, in the past decade. The prevalence of AmpC β-lactamase-producing *P. mirabilis* also increased significantly. Patient age and location were factors independently associated with the presence of ESBL and/or AmpC β-lactamase-producers. Cefotaxime was a better surrogate than ceftazidime for predicting the presence of ESBL and/or AmpC β-lactamases. Continuous surveillance on antimicrobial resistance and associated resistance mechanisms in *P. mirabilis* is warranted.

## Authors’ original submitted files for images

Below are the links to the authors’ original submitted files for images. Authors’ original file for figure 1 Authors’ original file for figure 2
