# Supplementary figures and images for: Antimicrobial susceptibilities of Proteus mirabilis: a longitudinal nationwide study from the Taiwan surveillance of antimicrobial resistance (TSAR) program
Source: BMC Infect Dis. 2014 Sep 5;14:486. doi: 10.1186/1471-2334-14-486 (PMC4162950; doi:10.1186/1471-2334-14-486)

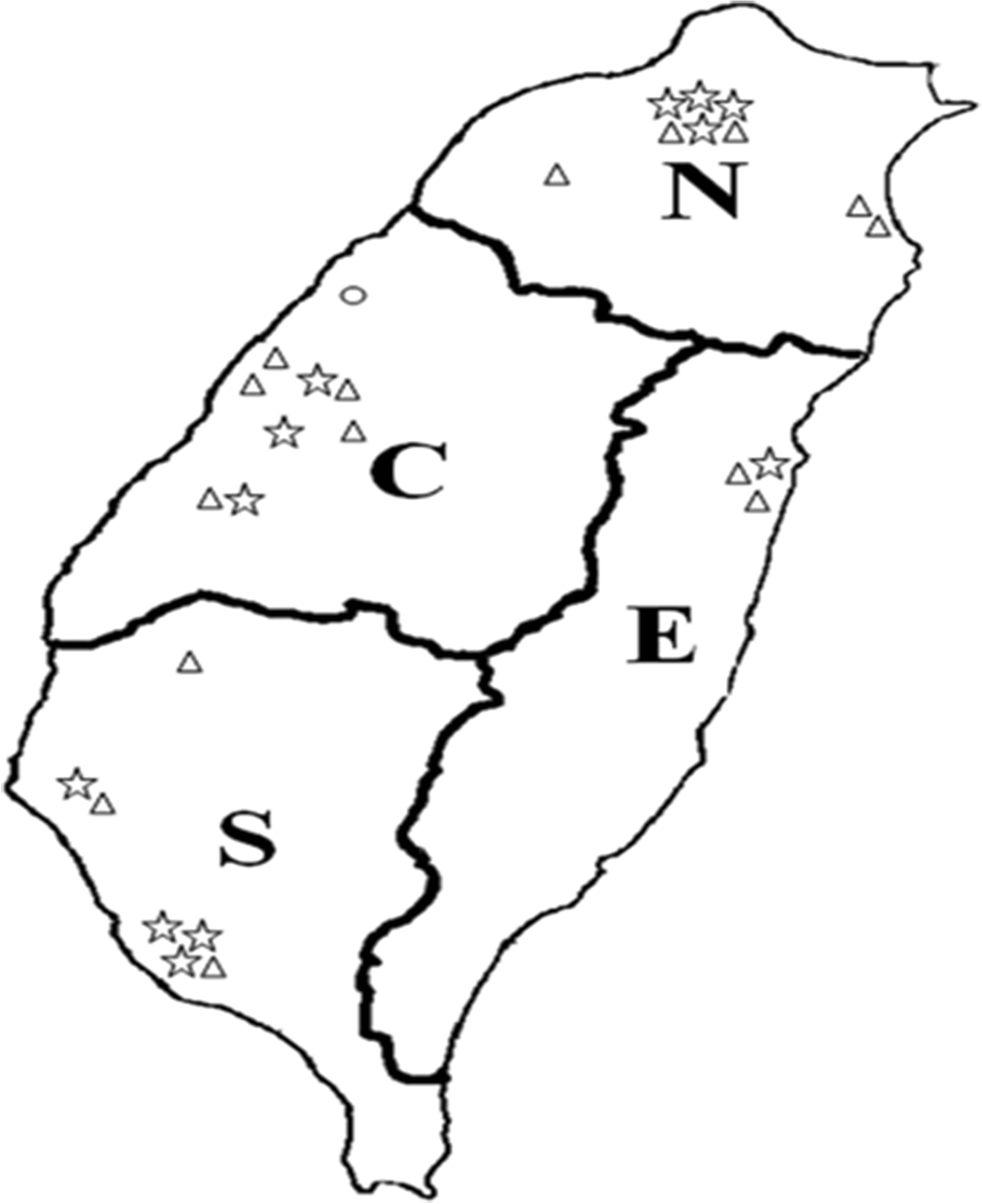

Supplement: Supplementary file 1 — Authors’ original file for figure 1 [file 12879_2014_3787_MOESM1_ESM.tif]

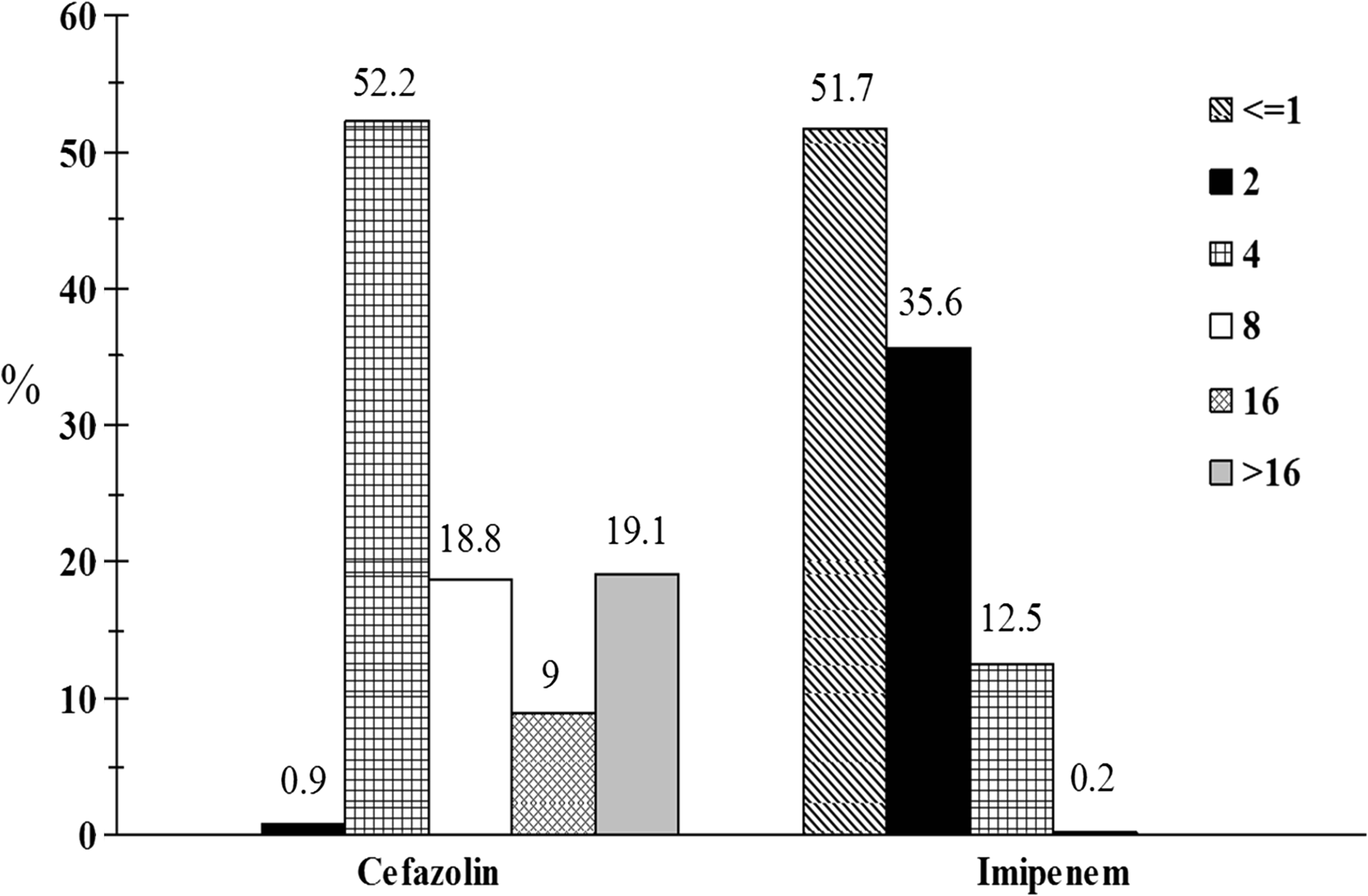

Supplement: Supplementary file 2 — Authors’ original file for figure 2 [file 12879_2014_3787_MOESM2_ESM.tif]
